# Muscle developmental defects in heterogeneous nuclear Ribonucleoprotein A1 knockout mice

**DOI:** 10.1098/rsob.160303

**Published:** 2017-01-11

**Authors:** Ting-Yuan Liu, Yu-Chia Chen, Yuh-Jyh Jong, Huai-Jen Tsai, Chien-Chin Lee, Ya-Sian Chang, Jan-Gowth Chang, Yung-Fu Chang

**Affiliations:** 1 Graduate Institute of Medicine, Kaohsiung Medical University, Kaohsiung, Taiwan, Republic of China; 2 Graduate Institute of Clinical Medicine, College of Medicine, Kaohsiung Medical University, Kaohsiung, Taiwan, Republic of China; 3 Department of Biomedical Science and Environmental Biology, College of Life Science, Kaohsiung Medical University, Kaohsiung, Taiwan, Republic of China; 4 Departments of Pediatrics and Clinical Laboratory, Kaohsiung Medical University Hospital, Kaohsiung, Taiwan, Republic of China; 5 Department of Biological Science and Technology, Institute of Molecular Medicine and Bioengineering, College of Biological Science and Technology, National Chiao Tung University, Hsinchu, Taiwan, Republic of China; 6 Institute of Biomedical Sciences, Mackay Medical College, New Taipei City, Taiwan, Republic of China; 7 Epigenome Research Center, China Medical University, Taichung, Taiwan, Republic of China; 8 Department of Laboratory Medicine, China Medical University, Taichung, Taiwan, Republic of China; 9 School of Medicine, China Medical University, Taichung, Taiwan, Republic of China; 10 Department of Medical Laboratory Science and Biotechnology, China Medical University, Taichung, Taiwan, Republic of China

**Keywords:** alternative splicing, embryonic development, hnRNP A1, knockout mice, muscle development

## Abstract

Heterogeneous ribonucleoprotein A1 (hnRNP A1) is crucial for regulating alternative splicing. Its integrated function within an organism has not, however, been identified. We generated hnRNP A1 knockout mice to study the role of hnRNP A1 *in vivo*. The knockout mice, *hnRNP A1*^−/−^, showed embryonic lethality because of muscle developmental defects. The blood pressure and heart rate of the heterozygous mice were higher than those of the wild-type mice, indicating heart function defects. We performed mouse exon arrays to study the muscle development mechanism. The processes regulated by hnRNP A1 included cell adhesion and muscle contraction. The expression levels of muscle development-related genes in *hnRNP A1*^+/−^ mice were significantly different from those in wild-type mice, as detected using qRT-PCR. We further confirmed the alternative splicing patterns of muscle development-related genes including *mef2c*, *lrrfip1*, *usp28* and *abcc9*. Alternative mRNA isoforms of these genes were increased in *hnRNP A1*^+/−^ mice compared with wild-type mice. Furthermore, we revealed that the functionally similar hnRNP A2/B1 did not compensate for the expression of hnRNP A1 in organisms. In summary, our study demonstrated that hnRNP A1 plays a critical and irreplaceable role in embryonic muscle development by regulating the expression and alternative splicing of muscle-related genes.

## 1. Introduction

Alternative splicing is the most critical posttranscriptional mechanism by which cells can generate a diverse repertoire of protein isoforms from a limited number of genes. This process plays a crucial regulatory role by altering the function, expression level and localization of gene products. It often occurs in response to the activities of key signalling pathways and is essential for development and differentiation [1]. Given its multifunctional role, alternative splicing has been shown to play an important role in human diseases such as amyotrophic lateral sclerosis, frontotemporal lobe dementia, spinal muscular atrophy, Alzheimer's disease and *LMNA*-related disorders [2,3].

Alternative splicing is modulated by *cis*-regulatory elements located within alternative exons or introns and by *trans*-acting splicing factors. *cis*-regulatory elements include enhancers and silencers. Splicing enhancers and silencers are either exonic or intronic, depending on whether they function from an exonic or intronic location. Serine/arginine (SR) proteins and heterogeneous ribonucleoprotein (hnRNP) protein families are well-known *trans*-acting splicing factors that promote or inhibit splice site recognition. Exonic enhancers bind SR proteins, which promote spliceosome assembly, whereas silencers include proteins of the hnRNP family to interfere with spliceosome construction and exon inclusion [4,5].

The hnRNP A1 protein is one of the most studied hnRNP proteins and is a key player in messenger RNA (mRNA) metabolism and biogenesis. It promotes the regulation of the transcriptional functions of target genes and the alternative splicing of more than 25 identified genes. Other functions regulated by hnRNP A1 include telomere maintenance, mRNA nuclear export, mRNA translation and turnover, and microRNA processing [6]. However, the role of hnRNP A1 in embryonic development and muscle differentiation *in vivo* is unclear.

Muscle was the first tissue studied for tissue-specific alternative splicing. To date, more than 1000 muscle-specific alternative splicing events have been reported [7]. Alternative splicing transitions also occur in the heart during postnatal development and during differentiation of the myogenic C2C12 cell line in mice [8]. These articles suggest that the hnRNP family plays a critical role in muscle-specific alternative splicing, so identifying the role of its major member, hnRNP A1, would be highly beneficial for the understanding of muscle-specific alternative splicing *in vivo*.

hnRNP A1 regulates numerous functions in cells. Its integrated function in an organism has not, however, been identified. In this study, we generated hnRNP A1 knockout mice to study the function of hnRNP A1 *in vivo*. We examined the phenotype and physiological functions of these mice and investigated the molecules regulated by hnRNP A1.

## 2. Results

### 2.1. Generation of hnRNP A1 knockout mice

**Figure 1.**
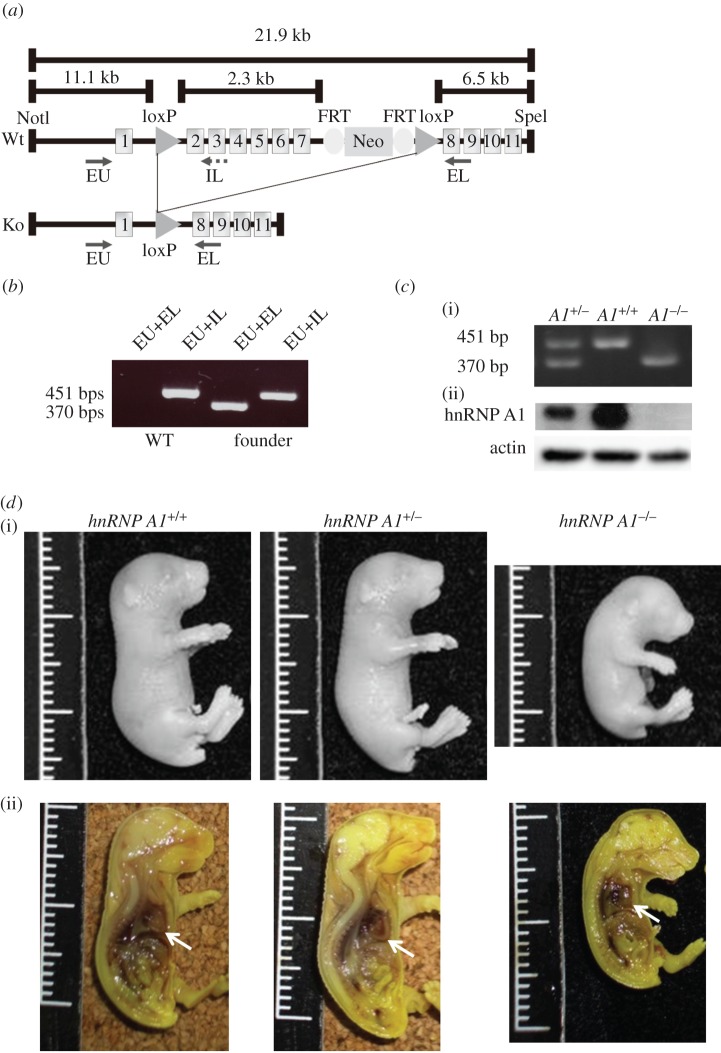
Generation of hnRNP A1 knockout mice. (*a*) Map of the *hnRNP A1* gene used in gene targeting. Exons 2–7 of the *hnRNP A1* gene were removed using Cre-loxP recombination. (*b*) Genotype of the *hnRNP A1*-targeted allele was detected using PCR. The primers employed to detect the *hnRNP A1*^+^ allele were EU and IL, with predicted products of 451 bp. The primers used to detect the *hnRNP A1*^−^ allele were EU and EL, with predicted products of 370 bp. (*c*) (i) Genotypes of the mice identified using PCR. (ii) hnRNP A1 proteins from the hearts of E18.5 mice were detected using Western blotting. β-actin was employed as a loading control. (*d*) Morphology (i) and gross anatomy (ii) of E18.5 *hnRNP A1* mutant and wild-type mice. The hearts of the mice are indicated by an arrow.

To identify the role of hnRNP A1 *in vivo*, we generated *hnRNP A1* knockout mice. The *hnRNP A1* gene is disrupted by deleting exons 2–7, a strategy illustrated in figure 1*a*. Polymerase chain reaction (PCR) was used to confirm that the founder mice were generated successfully (figure 1*b*). To generate homozygous *hnRNP A1* knockout mice, we intercrossed heterozygous *hnRNP A1*^+/−^ mice. The DNA and protein of *hnRNP A1* in the mice were detected using PCR and western blotting (figure 1*c*). The hnRNP A1 protein was not be detected in the mice when western blotting was used, indicating that hnRNP A1 knockout mice were successfully generated. The number of mice for each genotype was then calculated. Surprisingly, the number of knockout (*hnRNP A1*^−/−^) newborn mice (8/83, observation/total) was significantly lower than the expected value (20.75/83, expectation/total) according to Mendelian law, and these newborn mice died within 30 min. However, the number of heterozygous (*hnRNP A1*^+/−^; 57/83) and wild-type (*hnRNP A1*^+/+^; 18/83) newborn mice approximated our expectations (41/83, *hnRNP A1*^+/−^; 18/83, *hnRNP A1*^+/+^). These data indicated that some homozygous mice might have been embryonic lethal. To test this hypothesis, we acquired embryo 18.5 (E18.5) fetal mice by using Caesarean section. We obtained 82 fetal mice sourced from more than 10 litters. There were 22 wild-type, 43 heterozygous and 17 homozygous mice. These results were similar to our expectations (20.5 wild-type, 41 heterozygous and 20.5 homozygous mice), supporting our speculation on embryonic lethality in the hnRNP A1 knockout mice. To study the phenotype of hnRNP A1 knockout mice, we used a whole-mount section to perform haematoxylin and eosin (H&E) staining. The body lengths of the knockout mice were shorter than those of the wild-type and heterozygous mice. However, the body lengths of the heterozygous mice were similar to those of the wild-type mice. Although all organs were observed in the knockout mice, the hearts in these mice were round because of dilation of the ventricles (figure 1*d*).

### 2.2. Muscle development defects in hnRNP A1 knockout mice and zebrafish

**Figure 2.**
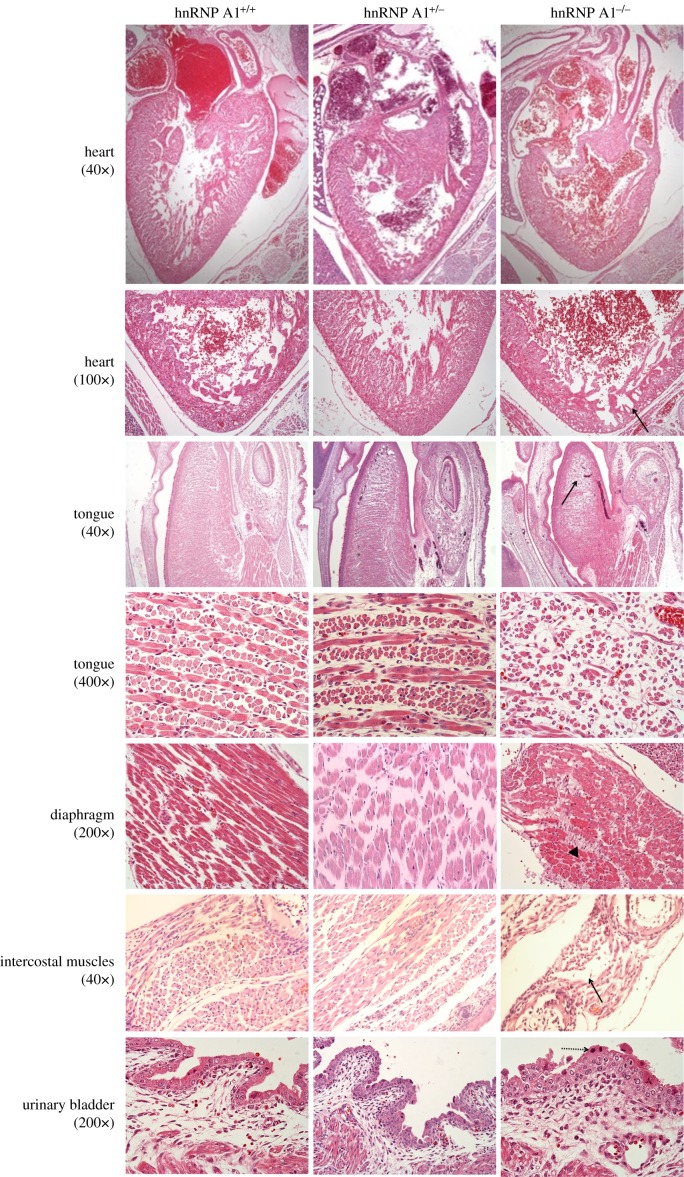
Muscle development defects in *hnRNP A1* mutant mice, with 40×, 100×, 200× and 400× representing the image magnification. The tissues of E18.5 mice were subjected to H&E staining. The solid arrow identifies myofibril hypoplasia in the heart, tongue and intercostal muscles. The arrowhead marks fibrous tissue infiltration in the skeletal muscle of the diaphragm. The dashed arrow identifies hyperplasia in urinary bladder transitional cells.

We discovered dilated cardiomyopathy with myofibril hypoplasia in the hearts of the hnRNP A1 knockout mice using histology (figure 2). The muscles in the tongues of the knockout mice were irregular compared with those of the wild-type and heterozygous mice. Hypoplasia of the tongue muscles and diaphragm skeletal muscles with fibrous tissue infiltration were observed in the hnRNP A1-depleted mice. Degeneration and hypoplasia were observed in the intercostal muscles of the knockout mice, and they also had urinary bladder defects with hyperplasia in the transitional cells.

To confirm the function of hnRNP A1 in embryonic development, we used zebrafish as a second model animal. When the morpholino (MO) antisense oligonucleotides of *hnRNP A1* were injected into the embryos of wild-type zebrafish at a concentration of 0.2 mM to specifically knockdown *hnRNP A1*, we discovered that approximately 77.7% (199 of 256 samples) of zebrafish embryos died prior to 24 h post fertilization (hpf), higher than the percentage of fish with scramble and PBS treatment (electronic supplementary material, figure S1*a*). When the *hnRNP A1* MOs were injected at a concentration of 0.15 mM into the embryos of transgenic line Tg(fli1:EGFP)^y1^ zebrafish, we discovered that 70.6% (173 of 245 samples) of injected embryos appeared abnormal in the post-dorsal axis at 72 hpf (electronic supplementary material, figure S1*b*, upper), although the defects were not lethal. Because the entire vascular system of embryos derived from the transgenic line was specifically tagged with a green fluorescent protein reporter, we observed the vasculature in embryos treated with 0.15 mM of *hnRNP A1* MO. The development of intersegmental vessels (ISVs) was discovered to be misdirected and appeared to have lateral asymmetry (electronic supplementary material, figure S1*b*, lower), suggesting that the abnormality in the development of the post-dorsal axis may cause abnormal ISV branching. Additionally, the heart in the *hnRNP A1*-knockdown embryos showed oedema (electronic supplementary material, figure S1*b*, lower). Furthermore, the expression levels of *hnRNP A1* were significantly decreased in hnRNP A1 knockdown zebrafish (electronic supplementary material, figure S1*c*). This demonstrated that hnRNP A1 plays a crucial role during embryonic development.

### 2.3. The defects of cardiac functions in hnRNP A1 heterozygous mice

**Figure 3.**
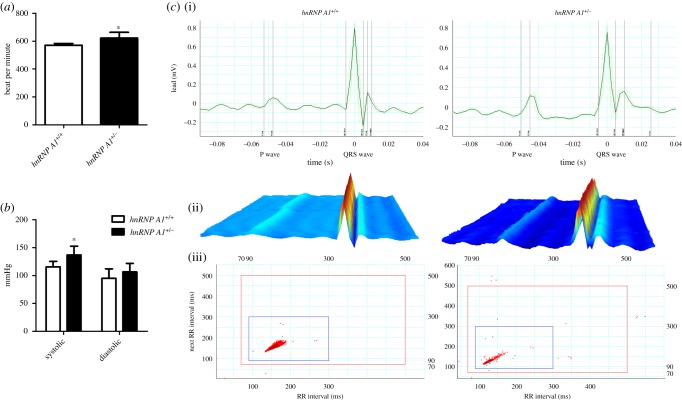
Heart function defects in *hnRNP A1* heterozygous mice. (*a*) Heart rate and (*b*) systolic and diastolic blood pressure of the mice. (*c*) Surface ECG of *hnRNP A1*^+/+^ and *hnRNP A1*^+/−^ mice aged six and eight weeks with the QRS complex and p wave (i), a three-dimensional graphic of the average ECG (ii) and a Poincaré plot displaying the variants in the RR interval (iii). Error bars represent standard deviation. *p < 0.05 compared with the wild-type mice. The results are summarized from observations of six mice.

The hnRNP A1 knockout mice had heart structure defects and the homozygous mice died within 30 min. Therefore, we investigated the heart function of the heterozygous mice even though they did not possess any obvious phenotype. The heart rates of the heterozygous mice were significantly higher than those of the wild-type mice (figure 3*a*), and the blood pressures, especially the systolic pressures, were also higher (figure 3*b*). Electrocardiography (ECG) was performed to examine the electrical activity of the heart. The RR interval, PR interval and P duration of the *hnRNP A1* heterozygous mice were less than those of the wild-type mice, although the difference was not statistically significant (figure 3*c*; electronic supplementary material, table S1). These data indicated that hnRNP A1 plays an essential role in cardiac function.

### 2.4. The expression of muscle-related genes was affected by hnRNP A1

**Figure 4.**
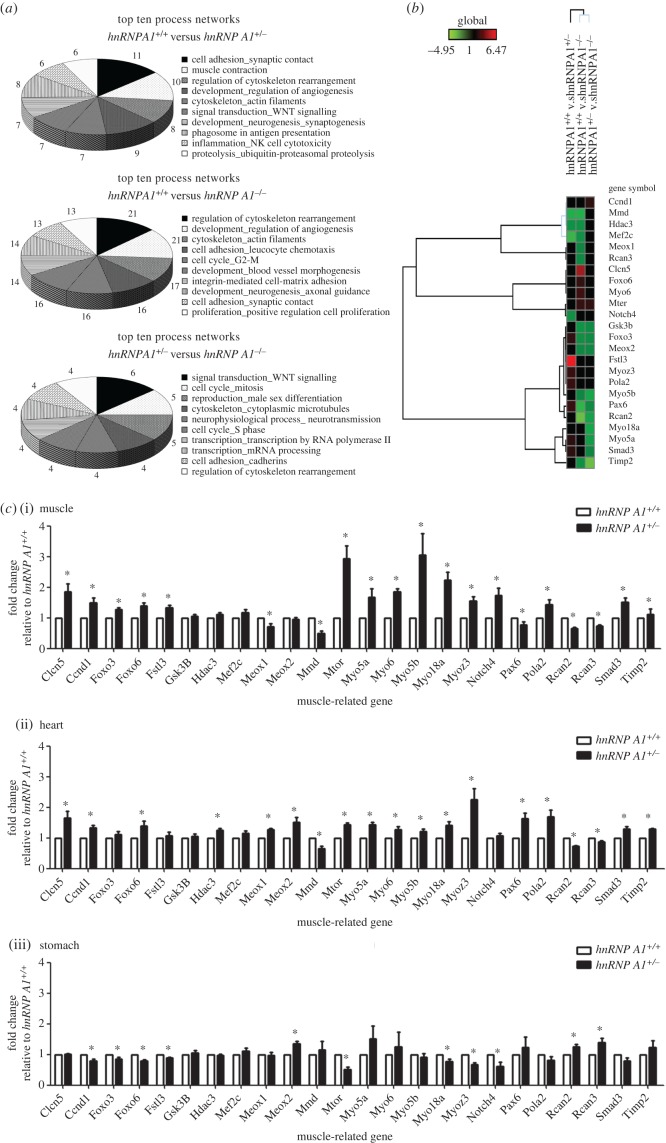
The differently expressed genes in hnRNP A1 defect mice. (*a*) The top 10 affected processes from the differentially expressed genes in the embryonic hearts of mice determined using microarray. The numbers represent gene numbers involved in the process. (*b*) Heat map of the list of muscle-related genes induced or repressed in the embryonic hearts of hnRNP A1 defect mice using microarray. Hierarchy clustering was used for gene grouping. (*c*) RNA levels of muscle-related genes in the heart (i), muscle (ii) and stomach (iii) analysed using qRT-PCR. The fold change was standardized using GAPDH mRNA levels and relative to *hnRNP A1*^+/+^ mice. Error bars represent standard deviation. *p < 0.05 compared with the wild-type mice. The results are summarized from observations of six mice.

To understand the underlying mechanism by which hnRNP A1 regulates expression of muscle-related genes, we used exon arrays to determine changes in global gene expression and alternative splicing. The gene expression patterns of the hnRNP A1 knockout and *hnRNP A1* heterozygous and wild-type mice were different (electronic supplementary material, figure S2). A total of 101 175 genes were analysed using microarrays. Among them, 6527 genes were upregulated whereas 6163 genes were downregulated in the heterozygous mice compared with the wild-type mice (electronic supplementary material, figure S2*a*); 5352 genes were upregulated whereas 6298 genes were downregulated in the knockout mice compared with the heterozygous mice (electronic supplementary material, figure S2*b*); and 4355 genes were upregulated whereas 4968 genes were downregulated in the knockout mice compared with the wild-type mice (electronic supplementary material, figure S2*c*). The common changed genes were analysed using MetaCore software and the data were enriched by process networks. The processes regulated by hnRNP A1 include cell adhesion, muscle contraction, regulation of cytoskeleton rearrangement and development regulation of angiogenesis. The 10 processes affected the most by hnRNP A1 are shown in figure 4*a*.

Because hnRNP A1 mice had muscle developmental defects, we chose for further study 24 muscle development-related genes (figure 4*b*) that were previously reported to be related to skeletal muscle development [9]. We confirmed the expression level of these 24 genes in heart, skeletal and stomach muscle tissues using real-time PCR. The expression level of most of these genes was significantly different in *hnRNP A1* heterozygous mice than in wild-type mice (figure 4*c*). These differently expressed genes included *mechanistic target of rapamycin* (*serine/threonine kinase*) (*mtor*), *myosin VI* (*myo6*) and *notch4*. We further studied the protein levels of these molecules using western blotting. The protein expression levels of mtor, myo6 and notch4 were increased in the hnRNP A1-defect mice and consistent with their mRNA expressions (electronic supplementary material, figure S3). These data indicated that hnRNP A1 modulated the expression level of the muscle development-related genes.

### 2.5. The alternative splicing of muscle-related genes was affected by hnRNP A1

**Figure 5.**
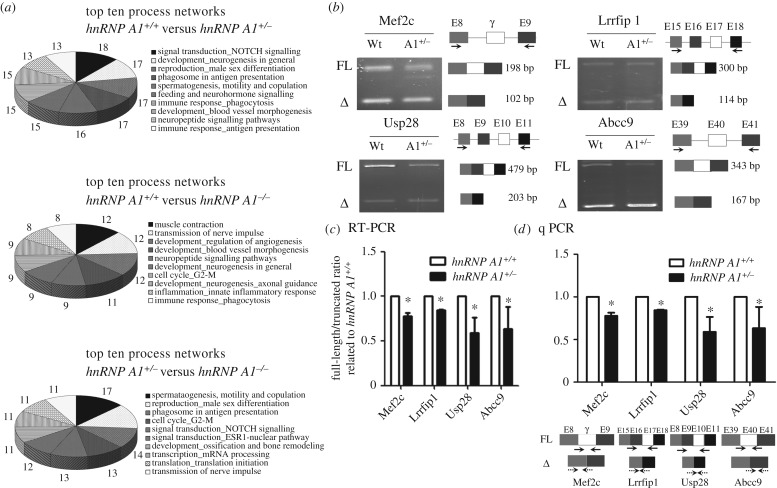
Alternative splicing genes in hnRNP A1 defect mice. (*a*) The top 10 affected processes from the alternative splicing genes in the embryonic hearts of hnRNP A1 defect mice determined using microarray. The numbers represent gene numbers involved in the process. (*b*) Various alternative splicing products of muscle-related genes in the hearts of mice analysed using qRT-PCR. The exon position, various alternative splicing products and RT-PCR primer positions are marked. The PCR products of full-length and truncated forms found using gel electrophoresis are indicated *hnRNP A1*^+/+^ (wt) and *hnRNP A1*^+/−^ (A1^+/−^). (*c*) Relative expression levels in (*b*) presented with the ratio (full/truncated length) relative to *hnRNP A1*^+/+^ mice. (*d*) The alternative splicing forms quantified using real-time PCR. The exon position, alternative splicing products and RT-PCR primer positions are presented in the lower part. Solid and dotted lines represent the primers for full-length and deleted mRNA, respectively. Error bars represent standard deviation. *p < 0.05 compared with the wild-type mice. The results are summarized from observations of six mice.

Alternative splicing is an essential function of hnRNP A1. We analysed the alternative splicing pattern of genes in the hnRNP A1-deficient mice using the microarray data. A total of 1 188 912 alternative splicing exons were screened in the microarrays. The processes regulated by hnRNP A1 in alternative splicing included neurophysiological and process transmission of nerve impulses, development regulation of angiogenesis, muscle contraction, signal transduction and neuropeptide signalling pathways, and development neurogenesis. The 10 most affected processes are presented in figure 5*a*. We further confirmed the alternative splicing pattern of muscle development-related genes according to the microarray data. Four genes with alternative splicing patterns were analysed using reverse transcription (RT) PCR: *myocyte enhancer factor 2C* (*mef2c*)*, leucine rich repeat in FLII interacting protein 1* (*lrrfip1*)*, ubiquitin specific peptidase 28* (*usp28*) and *ATP-binding cassette, sub-family C, member 9* (*abcc9*). These genes have been reported to have different splicing forms during muscle development [10–13]. The full length compared with the truncated forms of the *mef2c, lrrfip1, usp28* and *abcc9* genes was decreased in the *hnRNP A1* heterozygous mice compared with the *hnRNP A1*^+/+^ mice (figure 5*b*). The ratio of full to truncated length of each gene is shown in figure 5*c*. We also used real-time PCR to quantify each alternative splicing form of these genes (figure 5*d*). The ratios of the full to truncated length of each gene were decreased, which was consistent with the results of RT-PCR. These data indicated that hnRNP A1 regulated alternative splicing of muscle development-related genes *in vivo*.

### 2.6. hnRNP A2/B1 could not compensate for the protein expression of hnRNP A1

**Figure 6.**
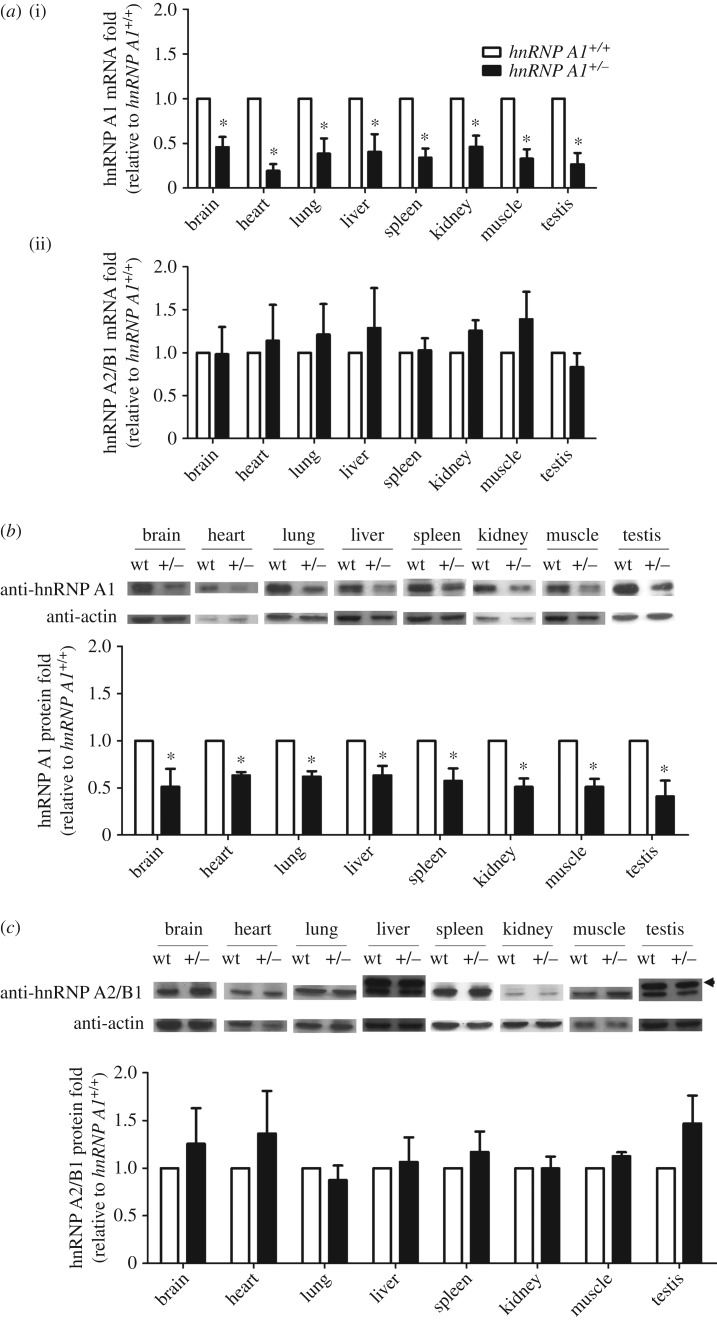
hnRNP A2/B1 did not compensate for the expression of hnRNP A1. (*a*) Total RNA of different tissues isolated and subjected to qRT-PCR to detect mRNA levels of *hnRNP A1* (i) and hnRNP A2/B1 (ii) in 18- to 20-week-old mice. The fold change was standardized using *gapdh* mRNA levels. Protein levels of (*b*) hnRNP A1 and (*c*) hnRNP A2/B1 (36/38 kDa) detected using western blotting. The arrowhead indicates hnRNP A2 (38 kDa). The fold change was standardized using β-actin protein levels. Error bars represent standard deviation. *p < 0.05 compared with the wild-type mice. The results are summarized from observations of nine mice.

hnRNP A2/B1 is a molecular homologue of hnRNP A1. A previous study has shown that hnRNP A2 compensated for the protein expression of hnRNP A1 in HeLa cells with hnRNP A1 deficiency [14]. We examined whether this compensation occurred *in vivo*. We detected the mRNA and protein expression levels of both hnRNP A1 and hnRNP A2/B1 in hnRNP A1 mutant mice. The levels of *hnRNP A1* mRNA in various organs of the heterozygous mice were lower than those observed in the wild-type mice (figure 6*a*, top). However, the levels of hnRNP A2/B1 mRNA in various organs of the heterozygous mice were comparable with those observed in the wild-type mice (bottom of figure 6*a*). We further detected levels of hnRNP A1 protein using western blotting. The protein levels in various organs of the *hnRNP A1* heterozygous mice were significantly lower than those observed in the wild-type mice (figure 6*b*). The levels of hnRNP A2/B1 protein in various organs of the heterozygous mice were similar to those observed in the wild-type mice (figure 6*c*). These data indicated that hnRNP A2/B1 did not compensate for the partial loss of hnRNP A1 expression.

In conclusion, the hnRNP A1 knockout mice had muscle defects in the heart, tongue, diaphragm and intercostal muscles, which ultimately caused death. The *hnRNP A1* heterozygous mice had heart function defects. The expression level of muscle development-related genes was different between hnRNP A1-deficient and wild-type mice. The different alternative splicing patterns of muscle development-related genes between hnRNP A1-deficient and wild-type mice were also demonstrated. Furthermore, the *in vitro* functionally similar hnRNP A2/B1 protein did not compensate for the expression and function of hnRNP A1. These data all indicate the crucial and irreplaceable functions of hnRNP A1 in an organism.

## 3. Discussion

Alternative splicing is a vital process in organisms [15]. Because hnRNP A1 is one of the key molecules in alternative splicing [16], understanding the effect of hnRNP A1 *in vivo* is crucial. In this study, we generated hnRNP A1 knockout mice to investigate the role of hnRNP A1 in embryonic development. We discovered that hnRNP A1 knockout mice had muscle differential defects (figure 2). Previous studies have shown that hnRNP K [17], hnRNP U [18] and tra2β [19] knockout mice possessed early embryonic lethality. Our results demonstrated that hnRNP A1 knockout mice were also embryonic lethal. The hnRNP A1 was previously reported to be upregulated in differentiating smooth muscle cells at E12.5 in mouse embryos [20]. We further demonstrated that *hnRNP A1* mutant mice possessed cardiac and skeletal muscle defects during embryonic development *in vivo*. The aggregation and abnormal distribution of hnRNP A1 were previously reported to correlate with oculopharyngeal muscular dystrophy [21] and sporadic inclusion body myositis [22]. We revealed that hnRNP A1 knockout mice had defects in the heart and skeletal muscles that caused their death (figure 2). These findings indicate that hnRNP A1 was critical for muscle development.

Abnormal alternative splicing in cardiac genes is associated with acquired and inherited heart diseases [23]. Defects in the splicing factor that are pertinent to regulating cardiac-related genes such as hnRNP H, hnRNP U, RBM24 and tra2β are known to cause heart failure [12,18,24,25]. Our results demonstrated a high heart rate and blood pressure in *hnRNP A1* mutant mice (figure 3), confirming the importance of splicing factor in the function of heart *in vivo*.

According to the microarray data, numerous genes were regulated by hnRNP A1 through gene expression or alternative splicing (figure 4). Analysis indicated that many of the genes that were affected by *hnRNP A1* participated in muscle functions. The expression levels of *mtor*, *myo6* and *notch4* were significantly changed in hnRNP A1 defect mice compared with those in wild-type mice (figure 4). The *mtor* gene is essential for skeletal muscle regeneration [26], cardiomyocyte survival and cardiac muscle function [27]. Oleanolic acid was previously shown to suppress hnRNP A1 expression and increase mtor expression [28]. The expression levels of the *mtor* gene increased in our hnRNP A1 defect mice, which was consistent with a previous study [26]. The *myo6* gene was reported to be involved not only in deafness, but also in mild symptoms of cardiac hypertrophy [29]. The myo6-binding partners of the FMRP and hnRNP proteins are involved in transport and nascent transcript maturation [30]. We demonstrated that expression of the *myo6* gene was significantly increased in the hnRNP A1 defect mice. The *notch4* gene was positively correlated with muscle oxidative phenotype [31], and low expression of *notch4* was identified during heart development [32]. Our data confirmed that notch4 expression was increased in hnRNP A1 defect mice, which might be a critical factor in abnormal heart development.

Previous studies demonstrated that there were different splicing forms of the *mef2c, lrrfip1, usp28* and *abcc9* genes during muscle development [10–13]. The full length of *mef2c* decreased in MHC-CELF transgenic mice, leading to cardiac hypertrophy [13]. Inclusion of the alternative exons 16 and 17 of *lrrfip1* activated by SRSF10 was found to be a muscle-specific event and essential for myoblast differentiation [10]. The alternative splicing forms of *usp28* and *abcc9* changed in *rbm24* defect mice, causing dilated atria [12]. The alternative splicing patterns changed in our study, indicating that hnRNP A1 is essential in the regulation of alternative splicing, especially during muscle development (figure 5).

The structure and function of hnRNP A2/B1 and hnRNP A1 are similar [33,34]. hnRNP A2 has been reported to compensate for the protein expression of hnRNP A1 in HeLa cells [14,35]. However, our results demonstrated that hnRNP A2/B1 did not compensate for hnRNP A1 in the hnRNP A1 knockout mice (figure 6). This might be because the regulation of hnRNP A2/B1 expression in response to decreased hnRNP A1 is much more complicated in an animal than in a cell. Therefore, hnRNP A1 has a unique function that cannot be compensated for hnRNP A2/B1 in organisms. The relationship between hnRNP A1 and hnRNP A2/B1 needs further investigation.

In summary, we generated hnRNP A1 knockout mice. These mice had muscle differential defects that caused death, indicating the critical role of hnRNP A1 in embryonic development. The low protein level of hnRNP A1 in *hnRNP A1* heterozygous mice resulted in cardiac function defects. We also demonstrated that hnRNP A1 regulated the expression and alternative splicing of muscle-related genes. Furthermore, hnRNP A2/B1 did not compensate for the expression of hnRNP A1. These findings highlight the critical and irreplaceable roles of hnRNP A1 in regulating the muscle development of organisms.

## 4. Material and methods

### 4.1. Animals

C57BL/6JNarl mice (National Laboratory Animal Breeding and Research Center, Taiwan) were used. All animals were housed in the KMU Laboratory Animal Center under a 12 L : 12 D (08.00/20.00) cycle with free access to food and water. The experiments used eight- to 15-week-old *hnRNP A1*^+/−^ heterozygous mice. The mice were sacrificed using carbon dioxide, after which tissues were harvested.

### 4.2. Generation of hnRNP A1 knockout mice

The *hnRNP A1* gene was knocked out by deletion of exons 2–7, resulting in frameshift and generating a premature translational stop codon in exon 8. To achieve deletion, a targeting vector was generated using a recombineering method [36]. A 21.9 kb mouse genomic DNA fragment containing introns 1–11 of the *hnRNP A1* gene was obtained from a bacterial artificial chromosome clone, bMQ-281N24 (Geneservice Ltd, Waltham, MA, USA), derived from 129 strains of mice. The *hnRNP A1* gene fragment was inserted between NotI and SpeI of plasmid pL253. This construct was then inserted into intron 2 with loxP from plasmid pL452 and into intron 7 with neo cassette from plasmid pL451. The final targeting construct contained a homologous 5′ long arm of 11.1 kb and 3′ short arm of 6.5 kb. The MC1-thymidine kinase (TK) gene at the end of the *hnRNP A1* gene served as a negative selection marker. This vector was confirmed using DNA sequencing. The correct construct was then electroporated into R1 hybrid embryonic stem (ES) cells and selected using both G418 (240 µg ml^−1^) and ganciclovir (2 µM) for neo and TK, respectively, as described in a previous study [37]. The genotypes of ES cells with homologous recombination were verified using southern blotting. The targeting ES cells were then injected into blastocysts of C57BL/6JNarl mice to generate chimeric mice. The chimeric mice were backcrossed with C57BL/6JNarl mice, and the first-generation offspring with an agouti coat colour served as the founder mice (F_0_). These mice were further backcrossed with C57BL/6JNarl mice to produce mice with a pure genetic background. A patent for these mice was issued by the United States Patent and Trademark Office (US Pat. No. 8,946,503, B2), and they are available from the Rodent Model Resource Center, Taiwan under accession no. RMRC13102.

### 4.3. Genotyping of hnRNP A1-deficient mice

Routine genotyping was performed using DNA from mouse tails by PCR. The primers used in PCR to identify the non-mutated sequence (*hnRNP A1*^+^) were Ex-A1U (EU) 5′-TATAGCGGGATGTGACGTGTTTTG-3′ paired with In-A1 L (IL) 5′-AATGAATCAACACCCCGCAACAAC-3′ and producing products of 451 bp. The primers used for identifying the deleted sequence (*hnRNP A1*^−^) were Ex-A1U (EU) paired with Ex-A1 L (EL) 5′-ACTGCACCCACAATGCTTTAAGAG-3′ and producing products of 370 bp. PCR was performed at 95°C for 10 min; 35 cycles of 95°C for 30 s, 58°C for 45 s and 72°C for 1 min; and 72°C for 7 min.

### 4.4. Whole-mount section with H&E staining

The morphology of mouse embryos from embryonic day E18.5 was analysed using a whole-mount section. Mouse embryos were briefly fixed in Bouin's solution (Sigma-Aldrich, Shanghai, China) for 12 h. The entire embryos were then treated with 80% ethyl alcohol, trimmed, dehydrated, embedded in paraffin, sectioned at 3 μm thickness, and stained with H&E.

### 4.5. Electrocardiography and blood pressure measurement

Before ECG was performed, mice were anaesthetized using 1% (v/v) isoflurane in oxygen at a rate of 1 l min^−1^. The PR and RR intervals were measured and recorded using a Dual Bio Amp (ADInstruments, Bella Vista, NSW, Australia) and PowerLab (ADInstruments). The records were analysed using Chart 5.0 (ADInstruments). Blood pressure and heart rate were measured under general anaesthesia using the BP-2000 Series II Blood Pressure Analysis System (Visitech Systems, Apex, NC, USA). The measurements were repeated 10 times per day.

### 4.6. Microarray analysis

The hearts of E18.5 mouse embryos were harvested for RNA microarray analysis. The RNA was extracted using TRIzol Reagent (Invitrogen, Grand Island, NY, USA). The quality of the RNA was analysed using an RNA 6000 Nano LabChip kit (Agilent Technologies, Massy, France) and a NanoDrop spectrophotometer (Thermo, Palaiseau, France). Total RNA integrity numbers were between 8 and 10. GeneChip Mouse Exon 1.0 ST arrays (Affymetrix, Santa Clara, CA, USA) were hybridized according to recommendations of Affymetrix using the Ambion WT Expression Kit protocol (Life Technologies, Grand Island, NY, USA) and Affymetrix labelling and hybridization kits. Mouse Exon microarrays were hybridized with 10 µg of labelled DNA. Raw data, transcript data and exon data were controlled using Expression Console Software (Affymetrix, Santa Clara, CA, USA) at the Institut Curie microarray core facility. The data were analysed using MetaCore software (Thomson Reuters, New York, NY, USA). The data discussed in this paper have been deposited in the National Center for Biotechnology Information's Gene Expression Omnibus (GEO) [38] and are accessible through GEO Series under access number GSE79076.

### 4.7. RNA extraction, RT-PCR and quantitative RT-PCR

Total RNA was isolated from tissues using TRIzol Reagent (Invitrogen). A total of 2 µg RNA was reverse-transcribed into cDNA using SuperScript III Reverse Transcriptase (Invitrogen). The quantitative RT-PCR (qRT-PCR) was performed using the Roche LightCycler 480 Real-Time PCR System using the SYBR Green System (Roche). The primers for real-time PCR were as follows: for *hnRNP A1*, forward 5′-TGG AAG CAA TTT TGG AGG TGG-3′ paired with reverse 5′- GGT TCC GTG GTT TAG CAA AGT-3′; for *hnRNP A2/B1*, forward 5′-AAG AAA TGC AGG AAG TCC AAA GT-3′ paired with reverse 5′-CTC CTC CAT AAC CAG GGC TAC-3′; and for *gapdh*, forward 5′-AAG AGG GAT GCT GCC CTT A-3′ paired with reverse 5′-TTG TCT ACG GGA CGA GGA AA-3′. PCR was performed at 95°C for 5 min and 40 cycles of 95°C for 10 s, 60°C for 20 s and 72°C for 5 s. A melting curve was then performed by heating to 95°C, subsequently cooling to 65°C for 60 s, and slowly reheating to 98°C at 0.11°C s^−1^ with continuous measurement, followed by a final cooling stage at 40°C for 10 s. The alternative splicing forms of muscle-related genes were detected using RT-PCR. The primers for each gene are listed in electronic supplementary material, table S2. PCR was performed at 95°C for 5 min and 30 cycles of 95°C for 30 s, 60°C for 45 s and 72°C for 60 s. The intensity of the signals was measured using LabWorks image analysis software (UVP, Upland, CA, USA).

### 4.8. Protein extraction and western blotting

Proteins were extracted from tissues using RIPA lysis buffer (50 mM Tris, pH8.0, 150 mM NaCl, 1 mM EDTA, 1% NP40, 1% sodium deoxycholate, 0.1% SDS) and separated using SDS-PAGE. After electrophoresis, the proteins were transferred onto nitrocellulose membranes (GE Healthcare, Chicago, IL, USA). The membranes were blocked with 5% skim milk solution in TBST (10 mM Tris pH 7.6, 150 mM NaCl, 0.1% Tween20) for 1 h. They were further treated with primary antibodies in TBST containing 1% skim milk solution for 1 h. After being washed three times with TBST, the membranes were then incubated with HRP-conjugated secondary antibodies for 1 h. Subsequently, the membranes were immersed in the ECL Plus substrate (GE Healthcare) for 5 min. The chemiluminescence signals were detected using X-ray film and analysed using LabWorks. The antibodies used in this experiment were anti-hnRNP A1 (9H10, SIGMA, St. Louis MO, USA; 1 : 5000 dilution), anti-hnRNP A2/B1 (DP3B3, SIGMA; 1 : 5000 dilution), anti-Actin (Santa Cruz Biotechnology, Inc., Santa Cruz, CA, USA; 1 : 5000 dilution), anti-tubulin 4a (GeneTex; 1 : 1000 dilution), anti-notch4 (St John's Laboratory, London, UK; 1 : 1000 dilution), anti-myo6 (St John's Laboratory; 1 : 1000 dilution), anti-mtor (St John's Laboratory; 1 : 1000 dilution), horse anti-mouse IgG-HRP (Cell Signalling; 1 : 5000 dilution) and donkey anti-goat IgG-HRP (Santa Cruz Biotechnology; 1 : 5000 dilution).

### 4.9. Statistical analysis

Statistical analysis of the data was performed using Microsoft Excel 2010 (Microsoft Corp., Redmond, WA, USA) and SPSS (IBM, Armonk, NY, USA). A Student's *t-*test was conducted to evaluate differences between groups. A probability of less than 0.05 was considered statistically significant after normalization.

## Supplementary Material

supplementary

## Data accessibility

All the data are available without restriction. Microarray data are accessible through GEO Series under access number GSE79076 (https://www.ncbi.nlm.nih.gov/geo/query/acc.cgi?acc=GSE79076). Mice in this study are available from the Rodent Model Resource Center, Taiwan under accession number RMRC13102 (http://www.nlac.narl.org.tw/RMRC/webc/html/data/show.aspx?ix=111&page=12&kind=M).
