# Supplementary material for: Muscle developmental defects in heterogeneous nuclear Ribonucleoprotein A1 knockout mice
Source: Open Biol. 2017 Jan 11;7(1):160303. doi: 10.1098/rsob.160303 (PMC5303281; doi:10.1098/rsob.160303)
Supplement: supplementary [file rsob160303supp1.pdf]

Supplemental material and method

Specific knockdown of HnRNP A1 in zebrafish embryos

Wildtype AB strain zebrafish and transgenic line Tg(fli1:EGFP)^{y1} [1] were maintained in aquaria according to standard procedures described by Westerfield et al. [2]. The experiments and treatments for these animals have been reviewed and approved by the National Taiwan University Institutional Animal Care and Use Committee (ethics approval number NTU-101-EL-78). Fluorescence signal was visualized with a fluorescent microscope (MZ FLIII, Leica). The hnRNP A1 antisense morpholino oligonucleotide (MOs) (5' GGAAACGAACCCAGGTCTCCCGCGT 3') and scramble MOs(5'CCTCTTACCTCAGTTACAATTTATA 3')(GeneTools, Philomath, OR, USA) were prepared at a stock concentration of 1 mM and diluted to the desired concentration (0.15 or 0.2 mM) before use. The hnRNP A1 MO oligonucleotides were injected into 1- to 2-cell stages of zebrafish embryos. The control group was injected with Phosphate-buffered saline (PBS).

References

1 Lawson, N. D., Weinstein, B. M. 2002 In vivo imaging of embryonic vascular development using transgenic zebrafish. *Dev Biol*. **248**, 307-318.

2 Westerfield, M. 2007 *The Zebrafish Book: A Guide for the Laboratory Use of Zebrafish (Danio Rerio)*. M. Westerfield.

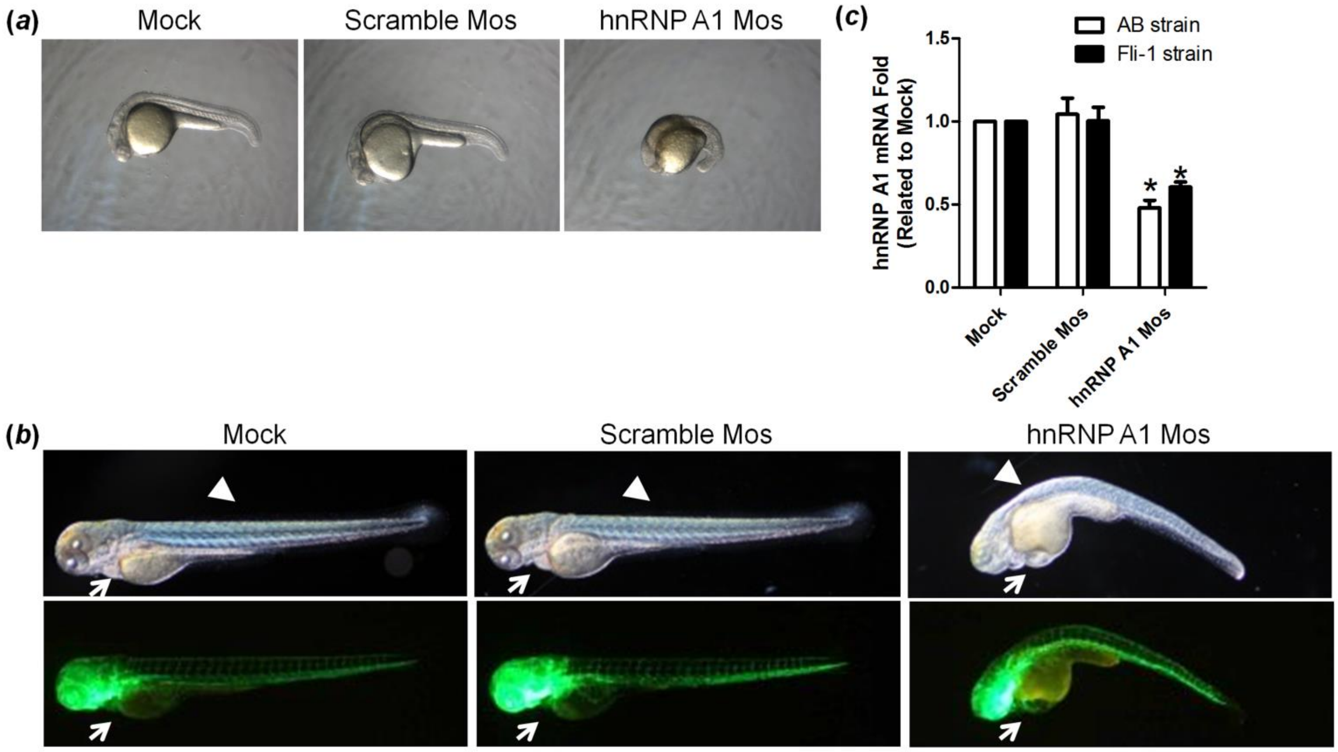

Figure S1

Figure S1. The knockdown of *hnRNP A1* using morpholino oligonucleotides in zebrafish. (a) The wild-type AB-strain zebrafish embryos injected with 0.2 mM *hnRNP A1* and scramble MOs or PBS for 24 hpf. (b) The transgenic line fli-1-strain zebrafish embryos injected with 0.15 mM *hnRNP A1* and scramble MOs or PBS for 72 hpf. The fish were examined using a fluorescent microscope (bottom). ◄ indicates the post-dorsal axis and the arrow represents the heart. (c) The mRNA expression levels of *hnRNP A1* in the zebrafish analysed using qRT-PCR. The fold change was standardised using *gapdh* mRNA levels relative to mock zebrafish. The white bar represents the *hnRNP A1* mRNA levels of wild-type AB-strain zebrafish (0.2 mM, 24 hpf). The black bar represents the transgenic line fli-1-strain zebrafish (0.15 mM, 72 hpf). Error bars represent standard deviation. (*) $P < 0.05$, compared with the mock zebrafish. The results are summarised from observations of six independent zebrafish. The mock group was injected with PBS.

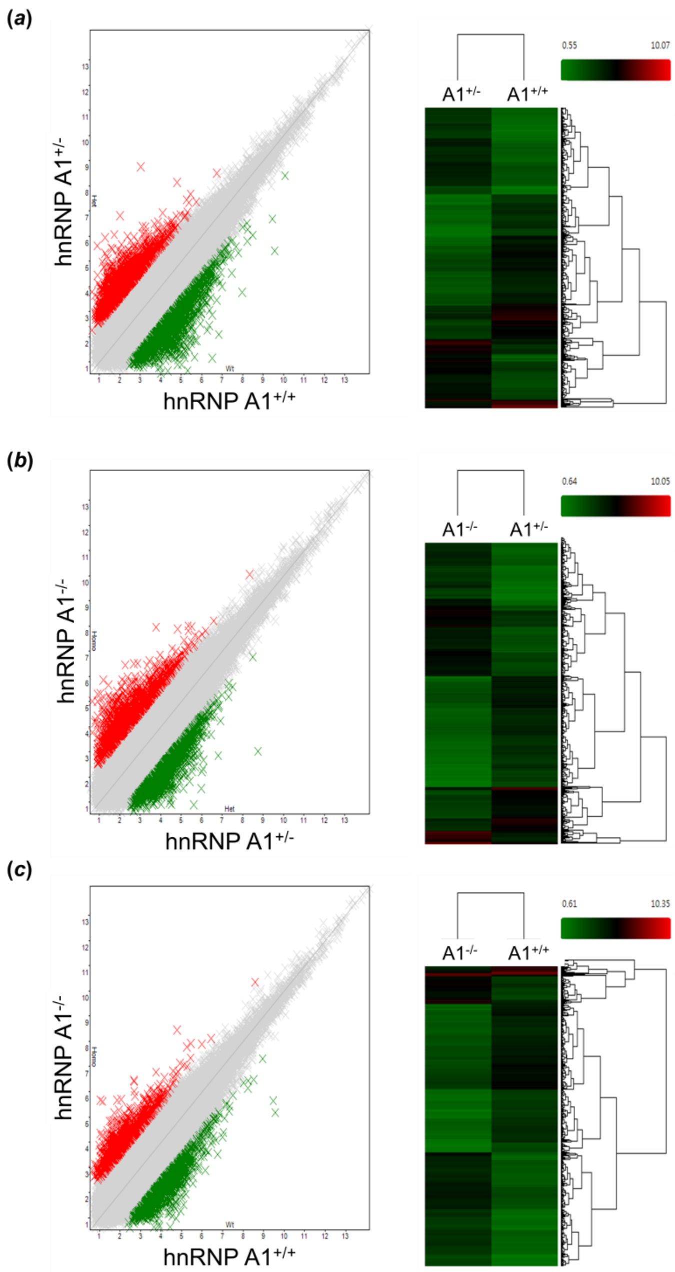

Figure S2

Figure S2. Identification of hnRNP A1–deficient mice through global gene expression. (a) Dot plot of the distribution of gene expression levels in *hnRNP A1* heterozygous and wild-type mice. (b) Dot plot of the distribution of gene expression levels in *hnRNP A1* heterozygous and homozygous mice. (c) Dot plot of the distribution of gene expression levels in *hnRNP A1* homozygous and wild-type mice. The green dots denote downregulated genes and the red dots denote upregulated genes. The filter criteria was fold change <(−3) or fold change > 3, $P < 0.05$.

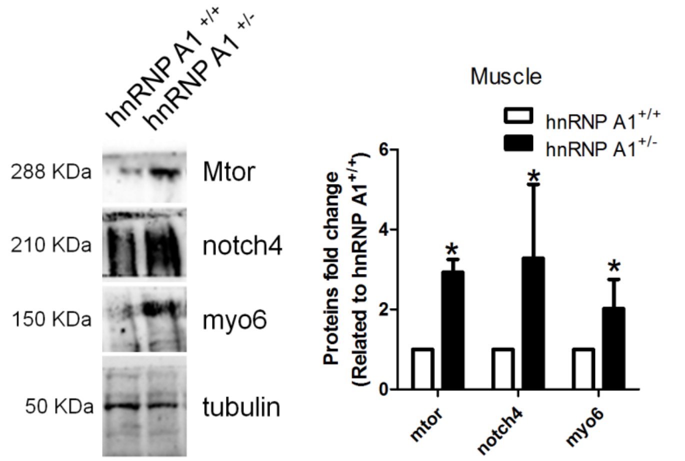

Figure S3

Figure S3. Expression levels of muscle-related proteins increased in muscle of hnRNP A1 defect mice. The mtor, notch4, and myo6 proteins were detected using Western blotting. The fold change was standardised using the internal control tubulin protein. Error bars represent standard deviation. (*) $P < 0.05$, compared with the wild-type mice. The results are summarised from observations of six mice.

Table S1 ECG intervals in mice

	hnRNP A1$^{+/+}$	*hnRNP A1*$^{+/-}$
RR Interval (ms)	199.6±59.26	138.4±16.29
PR Interval (ms)	54.4±1.21	47.8±3.82
P Duration (ms)	21.8±3.65	16.9±1.74
QRS Interval (ms)	11.7±0.54	11.1±1.21
QT Interval (ms)	23.9±0.42	22.4±3.8
QTc (ms)	60.3±0.7	60.7±12.5

The results are summarized from observations of 6 mice.

Table S2 Primer sequence

Gene	Forward Sequence	Reverse Sequence
Clcn5	GTG CCT GGT TAC ACA CAA CG	CTG TGC TAT ATG CTT TAA CAC ATC C
Ccnd1	TTT CTT TCC AGA GTC ATC AAG TGT	TGA CTC CAG AAG GGC TTC AA
Foxo3	GCT AAG CAG GCC TCA TCT CA	TTC CGT CAG TTT GAG GGT CT
Foxo6	AAG GAT AAA GGC GAC AGC AA	GTG TGC AGC GAC AGG TTG
Fstl3	AAT CAG CCT GCT AGG GTT CC	CTC CGT CGC AGG AAT CTT T
Gsk3b	GCA CTC TTC AAC TTT ACC ACT CAA G	CGA GCA TGT GGA GGG ATA AG
Hdac3	TTC AAC GTG GGT GAT GAC TG	TTA GCT GTG TTG CTC CTT GC
Mef2c	CCC ACG CAC TGA AGA AAA A	GGA TGG TAA CTG GCA TCT CAA
Meox1	AGA CGG AGA AGA AAT CAT CCA G	CTG CTG CCT TCT GGC TTC
Meox2	AAT CTA GAC CTC ACT GAA AGA CAG G	CTT GCT GTC CCC CTT TGA
Mmd2	GCT TTG CTA TGT GGT GAT GG	CCT GTC ATC AGC TCC CAG AT
Mtor	AGA AGA CAG CGG GGA AGG	GCA TCT TGC CCT GAG GTT C
Myo5a	CAG GCG AAG GAG ATG ACA G	TCA GGT CAA GCT CCA GTT GTT
Myo6	CCT GCA CCT TGG AAA TAT TGA T	GGG GCA GAT TTG TTC TTC AG
Myo5b	TCA CCA ACA CCG ACA AGA AG	GGA ACT TGT CTG GGT CCT TG
Myo18a	CAA GGT CAA GGA CCA GGA AG	CAT CTC CAG ACG CAG CTT G
Myoz3	GGG ACC TTG CTG AAC CAG	GAG ACA GCT CCT CTA TCA TTA GGT C
Notch4	GGA CCT GCT TGC AAC CTT C	CCT CAC AGA GCC TCC CTT C
Pax6	CAG TTC TCA GAG CCC CGT AT	CCA CCA AGC TGA TTC ACT CC
Pola2	CGA GCT GAG ATA CTT TGT GAA GG	CCA CTT GTC CTT TGG TGA GC
Rcan2	ATC TCA CCC CCT TCA TCT CC	CCA GCG TGC AGC TCA TAT T
Rcan3	CCA AGC AGT TCC TCA TCT CC	CGC GTG CAG TTC GTA TTT C
Smad3	TCC GTA TGA GCT TCG TCA AA	GGT GCT GGT CAC TGT CTG TC
Timp2	CGT TTT GCA ATG CAG ACG TA	GGA ATC CAC CTC CTT CTC G
AS-mef2c	TCC ACC TCG GCT CTG TAA CT	ATC TCG AAG GGG TGG TGG TA
AS-lrrfip1	GCA GTT CCA GTT TGC CG	GTG TCT GAA GTC TCT CCG
AS-usp28	TCC GAT GCT TGT CTT CTG AG	GAC AAG AAC ATG CTG AAG GC
AS-abcc9	GTA GTC ATG ACA GCC TTT GC	ACA AAC GAG GCA AAC ACT CC
Qmef2c-FL	TCA GTC AGT TGG GAG CTT GC	GGG TGG TGG TAC GGT CTC TA
Qmef2c-TR	TCC ACC TCG GCT CTG TAA CT	GGT ACG GTC TCC CAA CTG AC
Qlrrfip1-FL	GGA AGA GAT GCT CGA GGA AA	CCA TGT TTC TTC AAC TCC TCC T
Qlrrfip1-TR	GCA GTT CCA GTT TGC CG	TTT AGG ATT ATT CCA TGT TTC TCG
Qusp28-FL	AGC CTT GAG TGA GCA TAA TGA A	TGC CTT AAT AAG CCC TGC TTC
Qusp28-TR	TCC GAT GCT TGT CTT CTG AG	CAT GAA ATG CCT CAC TCA AGG
Qabcc9-FL	CCA TAG CTC ACC GTG TCT CTT	CCG ATG CAG AGA ATG AGA CA
Qabcc9-TR	ACC ATA GCT CAT CGG GTT CA	ACA AAC GAG GCA AAC ACT CC
Q-gapdh	AGC TTG TCA TCA ACG GGA AG	TTT GAT GTT AGT GGG GTC TCG
z-gapdh	TGC CAG TCA GAA CAT CAT CC	ACG GAA GGC CAT ACC AGT AA
z-hnrnp a1	GGC GGT AGA CGG TTT TAA GC	TCT GCC CTG TCA CCT CTC TC

Table S3. Metacore analysis of gene expression microarray data in process

a.hnRNP A1$^{+/+}$ versus hnRNP A1$^{+/-}$ process networks, enriched from 339 genes.

#	Networks	Total	p-value	In Data	Network Objects from Active Data
1	Cell adhesion_Synaptic contact	184	1.983E-04	11	Ephrin-A, Alpha-actinin, Syntaxin 1A, Synaptotagmin XI, CaMK II beta, Synaptotagmin, Ephrin-A5, Syntenin 1, CaMK II delta, PAK1, Alpha-actinin 1
2	Muscle contraction	173	5.104E-04	10	MYL4, Alpha-actinin, Syntaxin 1A, Calponin-3, MELC, SLMAP, PKC, CaMK IV, nAChR alpha,
3	Development_Regulation of angiogenesis	223	1.068E-02	9	Ephrin-A, CRK, DDAH1, Ephrin-A5, BTG1, PKC, PAK1, MALT1, PRK1
4	Cytoskeleton_Regulation of cytoskeleton rearrangement	183	1.010E-02	8	Alpha-actinin, CRK, MELC, Frabin, PKC-lambda/iota, PKC, PAK1, Alpha-actinin 1
5	Immune response_Phagosome in antigen presentation	243	4.550E-02	8	Alpha-actinin, CRK, PSMB1, MHC class I, HLA-C, HLA-B, PAK1, Alpha-actinin 1
6	Cytoskeleton_Actin filaments	176	2.513E-02	7	Alpha-actinin, CRK, ERBIN, MELC, TRIPs, PAK1, Alpha-actinin 1
7	Signal transduction_WNT signaling	177	2.582E-02	7	CaMK II beta, PTPA, CaMK II delta, Casein kinase I, PP2A regulatory, PP2A structural, Casein kinase I gamma-3
8	Development_Neurogenesis_Synaptogenesis	180	2.797E-02	7	Syntaxin 1A, Synaptotagmin XI, Synaptotagmin, KIS, P/Q-type calcium channel alpha-1A subunit, MJD (ataxin-3), nAChR alpha
9	Inflammation_NK cell cytotoxicity	164	5.201E-02	6	JAK1, MHC class I, HLA-H, HLA-C, HLA-B, PAK1
10	Proteolysis_Ubiquitin-proteasomal proteolysis	166	5.456E-02	6	SUMO-2, MUF1, PSMB1, NRDP1, NEDD4, MJD (ataxin-3)

b. hnRNP A1$^{+/+}$ versus hnRNP A1$^{-/-}$ process networks, enriched from 561 genes.

#	Networks	Total	p-value	In Data	Network Objects from Active Data
1	Cytoskeleton_Regulation of cytoskeleton rearrangement	183	5.274E-08	21	Plectin 1, SPTBN(spectrin1-4), MSN (moesin), 14-3-3 beta/alpha, ERM proteins, PARD6, Profilin II, Galpha(i)-specific amine GPCRs, ERK1 (MAPK3), Beta-fodrin, EPB41, Profilin, Frabin, MLCK, DOCK1, ERK1/2, PKC, PAK1, ERK2 (MAPK1), VIL2 (ezrin), 14-3-3
2	Development_Regulation of angiogenesis	223	1.512E-06	21	Ephrin-A, SPHK1, PKC-alpha, DDAH1, PDK (PDPK1), S2P, AGGF1, Ephrin-A5, Galpha(i)-specific peptide GPCRs, CBP, VEGFR-1, Ephrin-A receptors, ERK1 (MAPK3), Smoothened, FOXM1, IP3 receptor, ERK1/2, PKC, PAK1, ERK2 (MAPK1), PRK1
3	Cytoskeleton_Actin filaments	176	1.127E-05	17	Plectin 1, ERBIN, SPTBN(spectrin1-4), MSN (moesin), ERM proteins, MYLK1, Profilin II, LATS1, ERK1 (MAPK3), Beta-fodrin, EPB41, Profilin, MLCK, ERK1/2, PAK1, ERK2 (MAPK1), VIL2 (ezrin)
4	Cell adhesion_Leucocyte chemotaxis	205	2.653E-04	16	LPA1 receptor, CaMK II beta, PDK (PDPK1), Galpha(i)-specific peptide GPCRs, Galpha(q)-specific EDG GPCRs, ERK1 (MAPK3), VCAM1, Profilin, CaMK II gamma, PI3K cat class IB (p110-gamma), IP3 receptor, ERK1/2, Galpha(i)-specific EDG GPCRs, CaMK II delta, PAK1, ERK2 (MAPK1)
5	Cell cycle_G2-M	206	2.804E-04	16	Cyclin G, ESCO1, LATS2, UBE2C, SAM68, 14-3-3 beta/alpha, ANAPC4, Nucleolin, LATS1, Aurora-B, ERK1 (MAPK3), Cyclin G1, FOXM1, ERK1/2, Brca2, 14-3-3
6	Development_Blood vessel morphogenesis	228	8.617E-04	16	PDE3B, PKC-alpha, PDE, PDK (PDPK1), Galpha(i)-specific peptide GPCRs, Neuregulin 1, VEGFR-1, Galpha(q)-specific amine GPCRs, Galpha(i)-specific amine GPCRs, ERK1 (MAPK3), VCAM1, FOXM1, ERK1/2, Galpha(i)-specific EDG GPCRs, PAK1, ERK2 (MAPK1)
7	Cell adhesion_Integrin-mediated cell-matrix adhesion	214	3.451E-03	14	PKC-alpha, ALPHA-PIX, MSN (moesin), ERM proteins, PINCH, Profilin II, ERK1 (MAPK3), Profilin, MLCK, DOCK1, ERK1/2, PAK1, ERK2 (MAPK1), VIL2 (ezrin)
8	Development_Neurogenesis_Axonal guidance	230	6.521E-03	14	Ephrin-A, Ephrin-A5, ERM proteins, Ephrin-A receptor 7, cPKC (conventional), PP1-cat, SRGAP1, Ephrin-A receptors, Reelin, Semaphorin 4D, IP3 receptor, ERK1/2, PAK1, Plexin A4
9	Cell adhesion_Synaptic contact	184	2.463E-03	13	Ephrin-A, K(+) channel, subfamily J, Chapsyn-110, CaMK II beta, Ephrin-A5, Profilin II, Ephrin-A4, EPB41, Profilin, CaMK II gamma, IP3 receptor, CaMK II delta, PAK1
10	Proliferation_Positive regulation cell proliferation	221	1.132E-02	13	MLCP (cat), PDK (PDPK1), Galpha(i)-specific peptide GPCRs, VEGFR-1, FGF4, Galpha(i)-specific amine GPCRs, ERK1 (MAPK3), MLCK, PI3K cat class IB (p110-gamma), ERK1/2, PAK1, ERK2 (MAPK1), MTGR1

c. hnRNP A1$^{+/-}$ versus hnRNP A1$^{-/-}$ process networks, enriched from 237 genes.

#	Networks	Total	p-value	In Data	Network Objects from Active Data
1	Signal transduction_WNT signaling	177	1.109E-02	6	IP3 receptor, Tcf(Lef), Casein kinase I, TCF7 (TCF1), ERK3, Casein kinase I gamma-3
2	Cell cycle_Mitosis	179	4.139E-02	5	Stromalins 1/2, Importin (karyopherin)-beta, SAP97 (DLG1), SMC3, Karyopherin beta 1
3	Reproduction_Male sex differentiation	243	1.160E-01	5	PMEPA1, Olfactory receptor, TR4, SOX3, GFER
4	Cytoskeleton_Cytoplasmic microtubules	115	3.372E-02	4	ARHGEF2, SAP97 (DLG1), MAP2, Dynein, axonemal, heavy chains
5	Neurophysiological process_GABAergic neurotransmission	138	5.891E-02	4	Syntaxin 1A, IP3 receptor, IP3R1, PP1-cat
6	Cell cycle_S phase	149	7.373E-02	4	Stromalins 1/2, CHMP1A, SMC3, ERK3
7	Transcription_Transcription by RNA polymerase II	159	8.870E-02	4	TAF9L, TAFs, TAF5, TRAP95
8	Transcription_mRNA processing	160	9.027E-02	4	Nova1, SRPK1, SRPK2, NXF3
9	Cell adhesion_Cadherins	180	1.245E-01	4	Tcf(Lef), Casein kinase I, PTPRF (LAR), PP1-cat
10	Cytoskeleton_Regulation of cytoskeleton rearrangement	183	1.300E-01	4	ARHGEF2, Plectin 1, Dematin, Frabin

Table S4. Metacore analysis of alternative splicing microarray data in process.

a. hnRNP A1$^{+/+}$ versus hnRNP A1$^{+/-}$ process networks, enriched from 1040 genes.

#	Networks	Total	p-value	In Data	Network Objects from Active Data
1	Signal transduction_NOTCH signaling	236	7.544E-03	18	SFRP4, PDGF-A, p38gamma (MAPK12), PCNA, ARNT, TCF12, p70 S6 kinase2, FZD10, FBXW7, Skp2/TrCP/FBXW, WNT, p38 MAPK, c-Raf-1, FZD2, SMAD4, Frizzled, c-Fos, WNT6
2	Development_Neurogenesis in general	192	2.057E-03	17	SF1, POU3F3 (BRN1), CHRM, Myelin P0 protein, FBXW7, WNT, GFRalpha2, Galpha(q)-specific amine GPCRs, Galpha(i)-specific amine GPCRs, FZD2, DLX2, Frizzled, Calcipressin 1, POU class III, SOX14, WNT6, OTX2
3	Reproduction_Male sex differentiation	243	2.043E-02	17	SF1, UNR, PDGF-A, Olfactory receptor, HSF1, TR4, Histone H2, Histone H2A, p38 MAPK, MSK1, c-Raf-1, HOOK1, SMAD4, OCA2, PMEPA1, Translin, Histone H1
4	Immune response_Phagosome in antigen presentation	243	2.043E-02	17	p38gamma (MAPK12), TAP1 (PSF1), HLA-DQA1, Beta-2-microglobulin, FCGR3A, TAP, PLC-gamma 2, p38 MAPK, ERp72, TLR4, POR1, VAV-1, PLC-gamma, Derlin-2, PSMB4, TAP2 (PSF2),
5	Reproduction_Spermatogenesis, motility and copulation	228	2.349E-02	16	SF1, UNR, PDGF-A, HSF1, ODF3, TR4, BBS6, Histone H2, Histone H2A, MSK1, c-Raf-1, SMAD4, OCA2, RAD23B, Translin, Histone H1
6	Reproduction_Feeding and Neurohormone signaling	211	2.508E-02	15	Substance P extracellular region, IDE, TAC3, p70 S6 kinase2, Galpha(i)-specific peptide GPCRs, CRHR1, PLC-gamma 2, Substance P, c-Raf-1, PLC-gamma, Galpha(s)-specific CRF GPCRs, CYP19, c-Fos, TNF-alpha, PLC-beta
7	Immune response_Phagocytosis	222	3.708E-02	15	p38gamma (MAPK12), C/EBP, BAI1, MANR, FCGR3A, PLC-gamma 2, p38 MAPK, TLR4, APOA2, VAV-1, MRLC, PLC-gamma, HDL proteins, c-Fos, PLC-beta
8	Development_Blood vessel morphogenesis	228	4.515E-02	15	LDHA, ABCG2, ARNT, Galpha(i)-specific peptide GPCRs, CRHR1, Galpha(q)-specific nucleotide-like GPCRs, Thy-1, Galpha(q)-specific amine GPCRs, ANGPTL3, Galpha(i)-specific amine GPCRs, c-Raf-1, CEACAM1, Galpha(s)-specific CRF GPCRs, TRPC1, c-Fos
9	Signal transduction_Neuropeptide signaling pathways	155	1.040E-02	13	NPY, Substance P extracellular region, BAI1, TAC3, Galpha(i)-specific peptide GPCRs, CRHR1, Substance P, c-Raf-1, LEC2, NPY5R, SAP97 (DLG1), c-Fos, Galpha(s)-specific peptide GPCRs
10	Immune response_Antigen presentation	197	5.840E-02	13	CD8 alpha, CD8, TAP1 (PSF1), HLA-DQA1, AP-3 mu subunits, Beta-2-microglobulin, HLA-E, TAP, AP-3 beta subunits, CEACAM1, PSMB4, TAP2 (PSF2), TNF-alpha

b. hnRNP A1$^{+/+}$ versus hnRNP A1$^{-/-}$ process networks, enriched from 626 genes.

#	Networks	Total	p-value	In Data	Network Objects from Active Data
1	Muscle contraction	173	3.550E-03	12	CCKAR, VIP receptor 1, GCAP, Galpha(i)-specific peptide GPCRs, Sorcin, MaxiK alpha subunit, KCNQ1, Galpha(q)-specific amine GPCRs, Phospholemman, Galpha(q)-specific peptide GPCRs, C3aR, PKC
2	Neurophysiological process_Transmission of nerve impulse	212	1.684E-02	12	VIP receptor 1, MEK1/2, Galpha(i)-specific peptide GPCRs, Sorcin, Galpha(i)-specific metabotropic glutamate GPCRs, MaxiK alpha subunit, Serotonin receptor, mGluR4A, Galpha(i)-specific amine GPCRs, HTR1D, GABA-A receptor delta subunit, MEK2(MAP2K2)
3	Development_Regulation of angiogenesis	223	2.400E-02	12	CCL2, S2P, Galpha(i)-specific peptide GPCRs, Ephrin-A receptor 4, CRK, Ephrin-A receptors, Bcl-10, Galpha(q)-specific peptide GPCRs, CTGF, MEK2(MAP2K2), PKC, IGFBP7/8
4	Development_Blood vessel morphogenesis	228	5.815E-02	11	RBP-J kappa (CBF1), PDE, Galpha(i)-specific peptide GPCRs, CRK, Galpha(q)-specific amine GPCRs, ANGPTL3, Galpha(i)-specific amine GPCRs, Galpha(q)-specific peptide GPCRs, CTGF, MEK2(MAP2K2), IGFBP7/8
5	Signal transduction_Neuropeptide signaling pathways	155	3.132E-02	9	NPY, Urocortin, VIP receptor 1, MEK1/2, Galpha(i)-specific peptide GPCRs, LEC2, Galpha(q)-specific peptide GPCRs, NPY5R, MEK2(MAP2K2)
6	Development_Neurogenesis in general	192	9.385E-02	9	RBP-J kappa (CBF1), POU3F3 (BRN1), CHRM, ACM2, GFRalpha2, Galpha(q)-specific amine GPCRs, Galpha(i)-specific amine GPCRs, LHX5, POU class III
7	Cell cycle_G2-M	206	1.291E-01	9	CAP-G, MEK1/2, CAP-G/G2, Aurora-B, Brca1, PCTK1, TXNL4B, MEK2(MAP2K2), Kinase MYT1
8	Development_Neurogenesis_Axonal guidance	230	2.033E-01	9	NEFM, NGF, Ephrin-A receptor 3, Ephrin-A receptor 4, Ephrin-A receptors, NT-3, MEK2(MAP2K2), ITM2B, Plexin A4
9	Inflammation_Innate inflammatory response	180	1.373E-01	8	TLR5, TIRAP (Mal), C3a, C3, C3b, C3aR, SP-B, IRAK4
10	Immune response_Phagocytosis	222	2.919E-01	8	p22-phox, C3, MANR, CRK, C3b, ELMO2, iC3b, C3dg

c. hnRNP A1$^{+/-}$ versus hnRNP A1$^{-/-}$ process networks, enriched from 974 genes

#	Networks	Total	p-value	In Data	Network Objects from Active Data
1	Reproduction_Spermatogenesis, motility and copulation	228	8.274E-03	17	XRN2, PDGF-A, SHBG, SP7, HSF1, Tcfl5, BBS6, Insulin receptor, SPAG1, XIAP, SMAD4, SMCP, OCA2, PKC, CatSper1, Histone H1, ODF2L
2	Reproduction_Male sex differentiation	243	1.498E-02	17	PDGF-A, SHBG, Olfactory receptor, SP7, HSF1, AMH type II receptor, BOULE, Chk1, Insulin receptor, p38 MAPK, TLK2, XIAP, SMAD4, OCA2, PKC, CatSper1, Histone H1
3	Immune response_Phagosome in antigen presentation	243	9.746E-02	14	MBL2, p38gamma (MAPK12), TAP1 (PSF1), IKK-alpha, WASP, TAP, p38 MAPK, ERp72, TLR4, POR1, Derlin-2, PSMB4, TAP2 (PSF2), SEC61 gamma
4	Cell cycle_G2-M	206	6.217E-02	13	Histone H1.5, PDGF-A, MAD2b, p38gamma (MAPK12), Ceb1, Skp2/TrCP/FBXW, Chk1, p38 MAPK, RAD9, TXNL4B, Brca2, Histone H1, HUS1
5	Signal transduction_NOTCH signaling	236	1.378E-01	13	SFRP4, PDGF-A, p38gamma (MAPK12), ARNT, FBXW7, Skp2/TrCP/FBXW, WNT, p38 MAPK, ErbB4, WNT10B, SMAD4, MEK4(MAP2K4), WNT6
6	Signal transduction_ESR1-nuclear pathway	216	1.435E-01	12	CAPER, LCoR (MLR2), NRIF3, HSD17B1, NCOA2 (GRIP1/TIF2), IKK-alpha, XBP1, ErbB4, Adenylate cyclase, SMAD4, Adenylate cyclase type VIII, RBB2
7	Development_Ossification and bone remodeling	157	4.492E-02	11	PAX1, ALPL, SFRP4, SP7, FGF23, WNT, p38 MAPK, XIAP, SMAD4, SMAD6, Fetuin-A
8	Transcription_mRNA processing	160	5.031E-02	11	CAPER, SNRPD2 (SMD2), SFRS4, SF3B2, SF3B1, PABPC1, NXF3, RALY, CPSF5, TXNL4B,
9	Translation_Translation initiation	171	7.372E-02	11	RPS28, RPS12, BOULE, PABPC1, RPS15, RPL27A, eIF2B1, RPS2, eIF2S1, eIF6 (ITGB4BP),
10	Neurophysiological process_Transmission of nerve impulse	212	2.128E-01	11	GABA-A receptor gamma-2 subunit, Homer, NAV1.9, VIP receptor 1, Galpha(i)-specific peptide GPCRs, Serotonin receptor, Galpha(i)-specific amine GPCRs, RASGRF1, HTR1D, Homer 2, SAP97 (DLG1)
